# Barriers and facilitators for the sexual and reproductive health and rights of young people in refugee contexts globally: A scoping review

**DOI:** 10.1371/journal.pone.0236316

**Published:** 2020-07-20

**Authors:** Veronika Tirado, Josephine Chu, Claudia Hanson, Anna Mia Ekström, Anna Kågesten

**Affiliations:** 1 Department of Global Public Health, Karolinska Institutet, Stockholm, Sweden; 2 London School of Hygiene and Tropical Medicine, London, United Kingdom; 3 Department of Infectious Diseases, Karolinska University Hospital, Stockholm, Sweden; FHI360, UNITED STATES

## Abstract

**Background:**

The need to address sexual and reproductive health and rights (SRHR) in humanitarian settings is more urgent than ever, especially among young refugees. We conducted a scoping review to identify and synthesise the literature on perceived barriers and facilitators to SRHR among young refugees and interventions created to address their needs.

**Methods:**

We searched three databases (PubMed, Global Health and POPLINE) for peer-reviewed and grey literature published in English between January 2008 and June 2018 that reported on SRHR barriers, facilitators and interventions for young refugees aged 10 to 24 years. We extracted data using standardised templates and assessed the quality of studies according to study design. Data were charted using qualitative content analysis and organised in line with a socio-ecological framework (individual, social and community, institutional and health system, and structural).

**Findings:**

We screened 1,169 records and included 30 publications (qualitative, quantitative, and mixed methods) across 22 countries; 15 were peer-reviewed articles and 15 were from the grey literature. Twenty-two publications reported on young people in refugee camps or alternatives to camps (e.g. sustainable settlements), and eight referred to young refugees who had been resettled to a third country. We identified 19 sub-categories for barriers and 14 for facilitators at the individual, social and community, institutional and health system, and structural levels. No publications discussed the SRHR challenges faced by young homosexual, bisexual, transgender or queer refugees, or those living with HIV. Nine publications described interventions, which tended to focus on the provision of SRHR services and information, and the training of peers, parents, religious leaders and/or service providers.

**Conclusions:**

Findings highlight that while young refugees experience similar barriers to SRHR as other young people, many of these barriers are exacerbated by the refugee context. The limited number of publications and evidence on interventions underlines the immediate need to invest in and evaluate SRHR interventions in refugee contexts.

## Introduction

Universal access to sexual and reproductive health (SRH) is a fundamental human right that is central to achieving the 2030 Agenda, as emphasised in the Sustainable Development Goals related to good health and wellbeing, and gender equality [1]. Young people aged 10 to 24 years lie at the very heart of sustainable development, both as agents of change to achieve healthy, inclusive and stable societies, and because they are the ones most affected by impacts related to climate change, gender equality, poverty, conflict, and migration [2]. Yet, the sexual and reproductive health and rights (SRHR) of young people, who today account for 24% of the world’s population at 1.8 billion, are frequently overlooked [3, 4].

The need to address the SRHR of young people in humanitarian contexts is especially pressing. As of 2019, an estimated 70.8 million people had been forcibly displaced worldwide; of those, around 25.9 million were refugees and over half were under the age of 18 [5]. Evidence shows that refugee adolescents (10–19 years) and youth (15–24 years) often face challenges in accessing SRHR information and services due to the poor living conditions, inadequate sanitation and limited access to health services associated with conflict and displacement [6, 7], in addition to the stigma associated with sexual activity at a young age [8]. Experiences of forced migration may further impact young people’s power and agency to negotiate and make decisions related to their bodies and sexual relationships, thereby putting them at risk of sexual violence, HIV and other sexually transmitted infections (STIs), unintended pregnancies, unsafe abortions and preventable maternal deaths [9, 10]. For example, a systematic review found that refugee, migrant and internally displaced girls and young women in Africa commonly lack access to SRH information, while simultaneously facing high risk of gender-based violence (GBV) including sexual violence [8]. In addition, while in transit and upon reaching a host country, young refugees are met with new cultural, social and legal contexts [11, 12]. This is exemplified by a qualitative study which found that Afghan refugee women who had settled in California experienced cultural conflict between the traditional emphasis placed on family in their home country, versus the more liberal values held by their host country [13].

In order to improve SRHR and identify tailored interventions in the context of conflict and displacement, there is an urgent need to gather information to ensure that the needs of young refugees are respected, protected and fulfilled in line with international human rights standards [14, 15]. The available literature tends to report on the SRHR needs of young people [16–18], the SRHR needs of refugees in general [19–23], or the SRHR needs of a broader population such as refugee, migrant and internally displaced young women in Africa [8]; however, there is limited information available on the SRHR of young refugees [8, 24, 25]. To our knowledge, no study has compiled the global evidence on barriers and facilitators to SRHR for refugees aged 10 to 24 years.

To close this gap, we reviewed and synthesised the literature on perceived barriers and facilitators to SRHR among young refugees globally, and the interventions developed to address their needs. We focused on two central research questions:

1. What has been reported on the perceived barriers and facilitators to SRHR for young refugees worldwide?
2. What has been reported on current SRHR interventions that target young refugees?

For the purpose of the current review, we use the term ‘refugee’ to refer to any person who has undergone forced international migration, including asylum seekers. Different sub-populations of refugees (e.g. victims of trafficking) face different risks and challenges associated with their specific migration process and context–whether they are in transit, being resettled to a new country or voluntarily repatriating. Throughout this scoping review, we differentiate between refugees residing in camps or alternatives to camps, and refugees who have been resettled to a third country. In line with classifications used by UNHCR, the former is used in this review to group publications that report on refugees living in camps and spontaneous settlements, as well as those who have alternative arrangements, such as urban refugees living independently amongst host populations [26]. In refugee camps and spontaneous settlements, people tend to live in temporary shelters that can create challenges for their health, such as overcrowding and increased exposure to health hazards and violence. Alternatives to camps are diverse and depend on the host country’s context, but are intended to be sustainable solutions that ensure refugees are assisted and protected [26]. Also in line with classifications used by UNHCR, the latter grouping includes publications on refugees who have been granted permanent settlement in a third country, typically as part of UNHCR’s resettlement programme. Resettlement countries include the United States, Canada, Germany, the United Kingdom, Australia and the Nordic countries [27].

### Conceptual framework

We referred to a model by Kaufman et al. [28] when developing a socio-ecological framework to guide our review, including the data charting and organisation of results. Building on Bronfenbrenner’s ecological systems theory [29], our framework illustrates how a combination of individual (e.g. beliefs, behaviours), social and community (e.g. norms), institutional and health system (e.g. health services, education), and structural (e.g. laws, protection mechanisms) factors interact to shape health and wellbeing among young refugees.

## Methods

We conducted a scoping review, exploring both quantitative and qualitative publications, to achieve our aim. Scoping reviews have become increasingly popular in health research given their usefulness for mapping the range and nature of evidence in relation to complex topics and for identifying gaps in the literature [30, 31]. They are particularly relevant for examining emerging evidence on a topic and may act as precursors for more rigorous systematic reviews. We followed the guidelines for conducting a scoping review outlined by Arksey and O’Malley [32], Levac, Colquhoun and O’Brien [33] and the Joanna Briggs Institute [34]. The initial methods for screening, study selection and data charting were outlined in an unregistered protocol (S1 Appendix). The review was structured in line with the Preferred Reporting Items for Systematic Reviews and Meta-Analyses (PRISMA) extension for scoping reviews checklist (S2 Appendix).

### Eligibility criteria

We considered primary research studies of all designs as well as grey literature across all geographical regions; reviews were excluded as they did not add new primary data. In order to be included, studies had to meet the following inclusion criteria (S3 Appendix):

1. published in the English language;
2. published between 2008 and 2018, given the most recent increase in international migrants during this time period;
3. focus on young people between 10–24 years of age: ‘adolescents’ (10-19-year-olds), ‘youth’ (15-24-year-olds), or people across these age brackets [35];
4. focus on refugees (defined as those who have moved from one country to another to seek international protection, including asylum seekers and refugees who have fled their country of origin and are unable or unwilling to return because of well-established fear of persecution) [36]; and
5. report on perceived SRHR barriers, facilitators and/or interventions.

Accordingly, studies were excluded if they focused on internal migrants, such as rural-urban migrants, or migrant workers. Publications that presented data for young people plus other age groups, or refugees plus nationals, were only included if data were disaggregated by population.

### Information sources and search

We searched the peer-reviewed literature in two databases (PubMed and Global Health) and grey literature in one database (POPLINE) from 1 January 2008 to 15 February 2018. Our search strategy was built in four steps using free-text and controlled vocabulary (e.g. MeSH terms): 1) sexual and/or reproductive health and rights (e.g. "sexual health" OR "sexual rights" OR "reproductive health" OR "reproductive rights") AND barriers and/or facilitators including interventions (e.g. barrier* OR facilitator* OR intervention*) AND young people (e.g. adolescent* OR young OR youth) AND refugee (e.g. refugee* OR "asylum seekers") (S4 Appendix). The search strategy was adapted for each database (S5 Appendix). We also hand-searched the websites of six organisations working with SRHR in humanitarian contexts: UNFPA, WHO, UNICEF, Guttmacher Institute, Women’s Refugee Commission and Plan International, in addition to the reference lists from all included publications. The six international organisations were selected for hand searching as they were known to the authors as having engaged in research and advocacy for SRHR and/or in humanitarian settings. These particular organisations were selected to augment the collection of papers identified by the electronic searches.

After the initial database searches, we reviewed the abstracts to identify the most common and relevant keywords; these included ‘sociocultural’, ‘planning’, ‘teenage’ and ‘resettlement’. We then performed a second search across all included databases using the original search strategy plus the identified keywords. In addition, we conducted a third search in June 2018 to check for any newly published articles. In February 2020, we re-conducted the original PubMed search and removed filters that relied on publications having been indexed with MeSH terms by PubMed (e.g. Humans), to enable publications that had not yet been indexed to be included.

### Selection of sources of evidence

We imported all records into an online article management application [37] and removed all duplicates. Two authors (JC and VT) screened the titles and abstracts of all records using a 7-item screening process (S6 Appendix). Publications that passed the initial screening were subjected to full-text review. Disagreements at any stage were resolved by consensus or by a third member of the research team. We extracted data using a standardised 27-item template to capture information about the study characteristics (e.g. publication year, country of origin, host country, study population, study aim, design), reported barriers and facilitators, and intervention characteristics (e.g. setting, activities) if applicable (S7 Appendix).

### Data charting process

We conducted a charting process to sort and organise the included publications and elicit information and insights relevant to our review, focusing on the study region, setting, sample size and participants. Findings were analysed using qualitative content analysis and synthesised using a thematic, narrative approach [38, 39]. Specifically, we identified and extracted meaning units from the results sections of the publications, and employed an abstraction process to develop codes, sub-categories and categories (S8 Appendix). In the case of any overlap between grey literature and peer-reviewed publications, we only extracted information once to prevent duplication of the same data, and documented which publications originated from the same project. We initially used an inductive approach, where we examined the text without predetermined keywords or categories [40]. We then applied a more deductive approach, where we linked the codes that had emerged to the different levels of the socio-ecological model [28]. For qualitative studies, quotations were extracted to provide examples for each code. Sub-analyses by age and sex were not possible due to the lack of disaggregated data; we therefore reported findings for the broader group of young refugees.

### Critical appraisal of individual sources of evidence

We conducted a critical appraisal to assess the quality of all included primary studies. Two authors (JC and VT) independently assessed the quality of all included publications according to study design, using the Cochrane Collaboration Qualitative Methods Group’s criteria for the critical appraisal of qualitative research [41] and the Effective Public Health Practice Project’s Quality Assessment Tool [42] for any quantitative sections. These tools allowed us to examine the appropriateness of each study’s aim, methodology, design and reported findings; discrepancies about quality were discussed until a consensus was reached. Ethical approval was reported by 23 out of the 30 included publications. The remaining seven publications did not provide information on ethical approval. Of the five publications that were rated to be low quality in the qualitative appraisal, four publications did not provide information on ethical approval.

## Results

**Fig 1:**
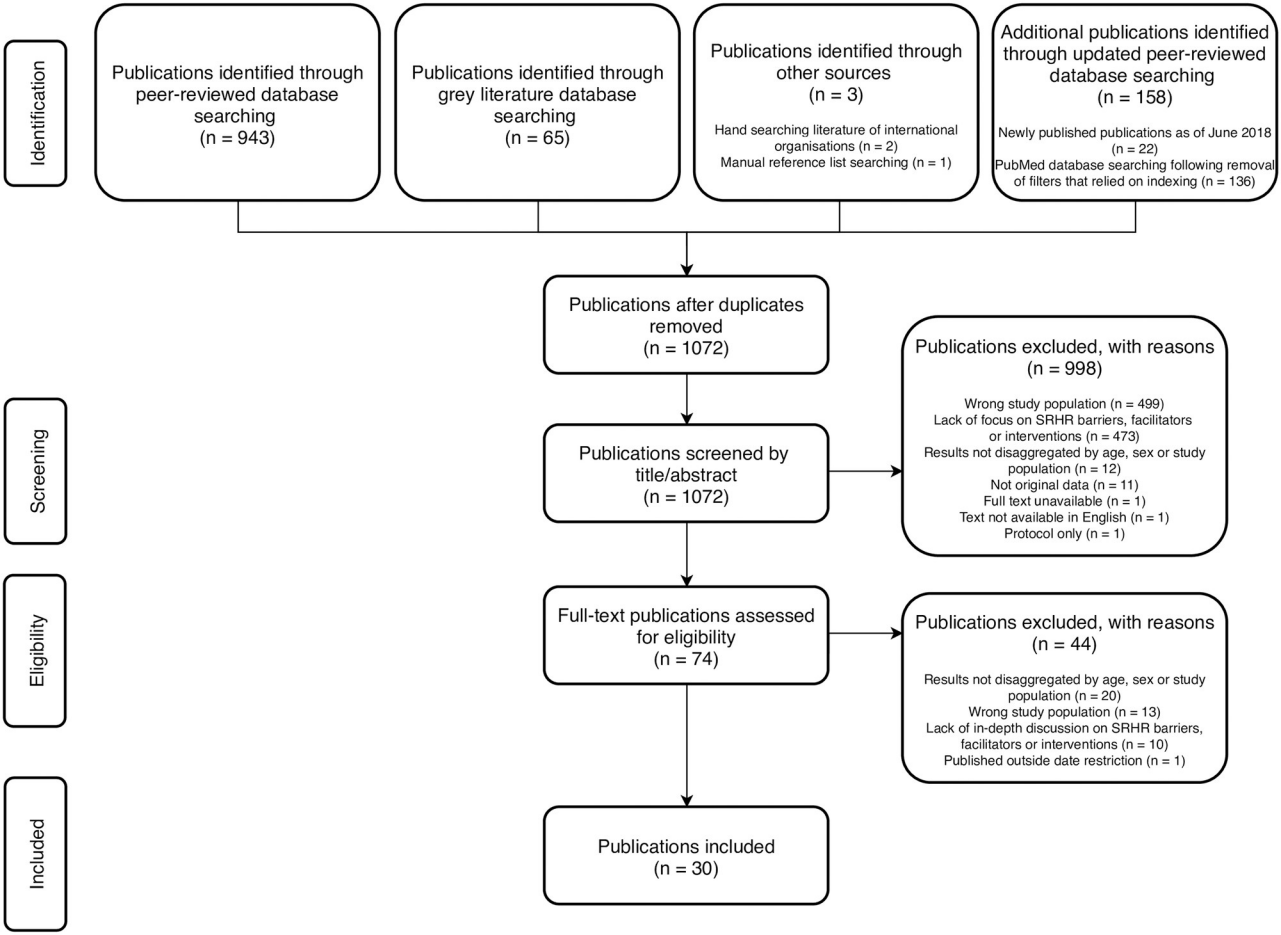
PRISMA flow diagram of the scoping review process.

Fig 1 shows the results from the searches, title and abstract screening and full-text review. In total, 1,169 records were identified across the peer-reviewed and grey literature. After the removal of duplicates, 1,072 records underwent title and abstract screening, resulting in 74 full-text publications to be assessed for eligibility. The main reasons for exclusion during the full-text reviews were lack of disaggregated results (20 publications), wrong study population (13 publications), lack of in-depth discussion on SRHR barriers, facilitators or interventions (10 publications), and published outside of the date restriction (1 publication). Finally, 30 publications [43–72] were included for data extraction and synthesis.

### Characteristics of the included publications

Table 1 shows the characteristics of the 30 included publications [43–72], of which 15 were from the grey literature [44, 45, 50, 51, 60–66, 69–72] and 15 were from peer-reviewed journals [43, 46–49, 52–59, 61, 67, 68]. A number of publications came from the same broader research project or study (Table 1). All of the included publications reported on barriers and/or facilitators to SRHR, while 9 out of the 30 publications [50, 51, 61, 63, 65, 68–71] also reported on interventions conducted to improve SRHR among young refugees.

**Table 1:** Summary of the included publications.

Author/s, year	Type	Country of origin	Host country	Study design	Age range in years (10–24; ≥ 80% under 24)	Sex	Refugee context
Benner et al., 2010 [43]	Peer-reviewed	Myanmar (Burma)	Thailand	Mixed methods	15–25	Males, females	Refugee camps
Connelly, 2011 [44]	Grey literature	Iraq	Jordan	Mixed methods	15–19	Males, females	Alternatives to camps
Cornier et al., 2011 [45] ^a^	Grey literature	Multiple countries including Somalia, the Democratic Republic of Congo, Iraq and Myanmar	Djibouti, Jordan, Kenya, Malaysia and Uganda	Mixed methods	10–19	Males, females	Refugee camps
Dean et al., 2016 [46]	Peer-reviewed	Sudan	Australia	Qualitative	16–24	Males, females	Resettled to a third country
DeJong et al., 2017 [47] ^b^	Peer-reviewed	Syria	Lebanon	Qualitative	10–16	Males, females	Alternatives to camps
Kingori et al., 2016 [48]	Peer-reviewed	Somalia	United States of America	Qualitative	18–25	Males, females	Resettled to a third country
Kågesten et al., 2017 [49] ^b^	Peer-reviewed	Somalia	Ethiopia	Quantitative	10–14	Males, females	Refugee camp
Lane, 2008 [50]	Grey literature	Sudan, the Democratic Republic of Congo and Burundi	Kenya and Tanzania	Qualitative	10–19	Males, females	Refugee camps
Lowicki-Zucca et al., 2013 [51]	Grey literature	Multiple countries including Rwanda, the Democratic Republic of Congo and Burundi	Uganda	Mixed methods	10–16	Males, females	Refugee settlement
Mantovani et al., 2013 [52]	Peer-reviewed	Minority ethnic groups from Southwest African, West and East African countries	United Kingdom	Qualitative	16–19	Females	Resettled to a third country
McMichael et al., 2009 [53] ^c^	Peer-reviewed	Multiple countries including Iraq, Afghanistan, Myanmar (Burma), Sudan and Liberia	Australia	Qualitative	16–25	Males, females	Resettled to a third country
McMichael et al., 2010 [54] ^c^	Peer-reviewed	Multiple countries including Iraq, Afghanistan, Myanmar (Burma), Sudan, Liberia and Ethiopia	Australia	Qualitative	16–25	Males, females	Resettled to a third country
Ngum Chi Watts et al., 2014 [55] ^d^	Peer-reviewed	Ethiopia, Sudan, Liberia, Burundi and Sierra Leone	Australia	Qualitative	17–30	Females	Resettled to a third country
Ngum Chi Watts et al., 2015 [56] ^d^	Peer-reviewed	Ethiopia, Sudan, Liberia, Burundi and Sierra Leone	Australia	Qualitative	17–30	Females	Resettled to a third country
Ngum Chi Watts et al., 2015 [57] ^d^	Peer-reviewed	Ethiopia, Sudan, Liberia, Burundi and Sierra Leone	Australia	Qualitative	17–30	Females	Resettled to a third country
Okanlawon et al., 2010 [58]	Peer-reviewed	Multiple countries including Liberia, Sierra Leone and the Democratic Republic of Congo	Nigeria	Mixed methods	10–24	Males, females	Refugee camp
Ortiz-Echevarria et al., 2017 [59] ^b^	Peer-reviewed	Somalia and Myanmar	Ethiopia	Qualitative	10–16	Males, females	Refugee camp
Paik, 2012 [60]	Grey literature	The Democratic Republic of Congo	Tanzania	Qualitative	10–16	Males, females	Refugee camp
Plan International, 2018 [61]	Grey literature	Burundi	Tanzania	Qualitative	10–19	Females	Refugee camp
Schulte et al., 2012 [62]	Grey literature	Somalia	Ethiopia	Qualitative	10–16	Males, females	Refugee camp
Tanabe, 2014 [63] ^e^	Grey literature	Bhutan	Nepal	Qualitative	15–19	Males, females	Refugee camps
Tanabe, 2014 [64] ^e^	Grey literature	Multiple countries including Somalia	Kenya	Qualitative	15–19	Males, females	Refugee camp
Tanabe et al., 2012 [65]	Grey literature	The Democratic Republic of Congo	Rwanda	Qualitative	10–19	Males, females	Refugee camp
Tanabe et al., 2014 [66] ^e^	Grey literature	Multiple countries including the Democratic Republic of Congo	Uganda	Qualitative	15–19	Males, females	Refugee settlements
Tanabe et al., 2015 [67] ^e^	Peer-reviewed	Multiple countries including Somalia, South Sudan and Ethiopia	Kenya, Nepal and Uganda	Qualitative	15–19	Males, females	Refugee camps
Tanabe et al., 2017 [68] ^a^	Peer-reviewed	Multiple countries including Somalia, the Democratic Republic of Congo, Iraq and Myanmar	Bangladesh, Djibouti, Jordan, Kenya, Malaysia and Uganda	Mixed methods	15–19	Males, females	Refugee camps
Tanner et al., 2017 [69]	Grey literature	Sudan and South Sudan	Ethiopia	Mixed methods	13–19	Females	Refugee camps
Turkmen Sanduvac, 2017 [70]	Grey literature	Burundi	Tanzania	Mixed methods	10–19	Females	Refugee camp
United Nations High Commissioner for Refugees, 2008 [71]	Grey literature	Multiple countries including Angola, Rwanda, Namibia, the Democratic Republic of Congo and Burundi	Zambia, Malawi, Botswana, Mozambique, Zimbabwe and Namibia	Qualitative	10–17	Males, females	Refugee camps
Women’s Refugee Commission et al., 2014 [72] ^b^	Grey literature	Multiple countries including Somalia, Syria and Myanmar	Ethiopia and Lebanon	Mixed methods	10–14	Males, females	Refugee camps

^a, b, c, d, e^ These publications originated from the same project or study, respectively.

Twenty publications employed a qualitative study design, nine employed mixed methods, and one employed a quantitative design. Study settings included, but were not limited to, Australia (6 publications) [46, 53–57], Kenya (5 publications) [45, 50, 64, 67, 68], Uganda (5 publications) [45, 51, 66–68], Ethiopia (5 publications) [49, 59, 62, 69, 72], and Tanzania (4 publications) [50, 60, 61, 70]. Sample populations included, but were not limited to, refugees from Sudan, the Democratic Republic of Congo, Somalia, Burundi, Iraq and Bhutan.

Twenty-three publications reported on studies conducted with both males and females, while seven were specific to females; no publications referred only to young male refugees. Half (15 publications) described studies with youth aged 15 years and above, and the other half also included younger adolescents (10 to 14 years); however, very few presented results disaggregated by age. In terms of the migration context, we found that 22 publications focused on young people living in refugee camps or alternatives to camps [43–45, 47, 49–51, 58–72] and 8 publications referred to young refugees who had been resettled to a third country [46, 48, 52–57].

During the critical appraisal of the qualitative content, 10 publications were rated as high quality, 15 publications were rated as moderate quality, and 5 publications were rated as low quality in line with the Cochrane Collaboration Qualitative Methods Group’s criteria (S9 Appendix). Of the 11 publications that also reported quantitative data (including mixed methods), none were rated as strong against the Effective Public Health Practice Project’s Quality Assessment Tool criteria, two publications were rated as moderate, and nine publications were rated as weak.

### Barriers and facilitators for the SRHR of young people in refugee contexts

**Fig 2:**
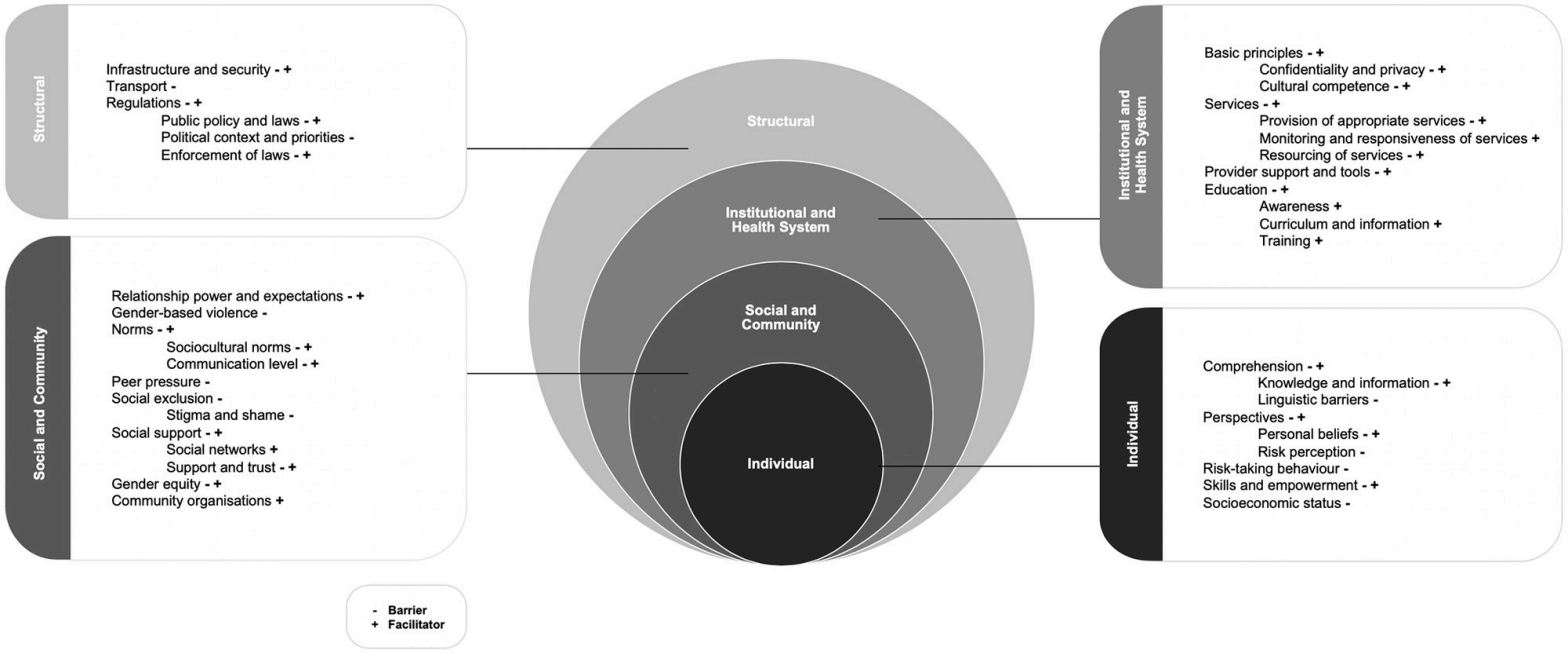
Socio-ecological framework showing barriers and facilitators for the SRHR of young refugees.

Fig 2 presents the identified barriers and facilitators in line with the different levels of the socio-ecological framework. Out of the 30 publications, 27 referred to the individual level (e.g. comprehension, perspectives, skills and empowerment), all 30 referred to the social and community level (e.g. relationship power and expectations, GBV, norms), 27 referred to the institutional and health systems level (e.g. basic principles, services, provider support, education), and 23 referred to the structural level (e.g. infrastructure and security, policies and laws, enforcement of laws). For the purpose of clarity, we differentiate between studies conducted with young refugees in camps or alternatives to camps, versus those conducted with young refugees who had been resettled to a third country.

Tables 2 and 3 each present an overview of the barriers and facilitators, respectively, arranged in line with each level of the socio-ecological framework, along with quotations from the included primary studies.

**Table 2:** Barriers to SRHR for young refugees.

Category	Sub-category	Code	Number of studies	References	Quotation
**Individual**	Comprehension	Knowledge and information	17	[43, 44, 46, 47, 49, 52–58, 61, 63, 67, 68, 71]	“Some were unsure whether using contraceptives would affect their ability to have children later on in life. This lack of understanding acted as a deterrent to using contraceptives for some” [56]
					“A few [people] indicated that knowledge of others’ experiences of crude abortions in refugee camps was a deterrent to aborting the baby” [57]
		Linguistic barriers	3	[51, 53, 64]	“Language barriers, both in terms of the diversity of languages spoken in the camp, as well as the lack of sign interpreters, were also raised by several groups of participants” [64]
	Perspectives	Personal beliefs	5	[43, 47, 51, 58, 67]	"If I should force him to use it [condom], he may leave me and go for another girl and I don’t want to lose him because he really helps me by giving me money” [58]
		Risk perception	4	[46, 51, 54, 68]	“It is widely assumed that carriers of sexually transmitted infections (STI) can be identified by their reputation and behaviour and ‘risky types’ and thereby avoided” [54]
	Risk-taking behaviour		7	[43, 45, 51, 58, 60, 62, 71]	“Some of the interviewed girls were known to exchange sexual favours to survive or meet basic material needs, at times for money, food items and other commercial goods, depending on availability and need” [60]
	Skills and empowerment		3	[60, 63, 67]	“Girls also emphasize that lack of personal, individual empowerment plays a role in their exposure to gender-based violence. Many girls say they lack life skills that they feel would help them better protect themselves. Many feel they need to be bolder and more assertive about saying no to unwanted advances” [60]
	Socioeconomic status		7	[43, 51, 52, 56, 67, 69, 70]	“The informants’ social positioning limited their social contacts with the outside world, which in turn had an impact on their help seeking behaviour and the options that were made available to them” [52]
**Social and Community**	Relationship power and expectations		5	[51, 54, 56, 58, 60]	“Some teachers use corporal punishment, including for ‘minor offences’ and, at times, for refusing sexual advances” [60]
	Gender-based violence		14	[43, 44, 47, 51, 59–64, 66, 69, 71, 72]	“Children reported serious violence against them, centring mainly around sexual violence: harassment and abuse of girls by groups of teenage boys, rape, transactional sex with older men–sometimes encouraged by parents–and marriages forced by parents in exchange for goods or money” [71]
					“Adolescent boys with mild intellectual impairments additionally suggested that ‘she will be locked in the house’ and ‘she might be circumcised again.’ Such feedback suggests limited autonomy in sexual and reproductive health decision making for unmarried women and adolescent girls with disabilities in particular” [64]
	Norms	Sociocultural norms	23	[43–47, 49, 51, 53–59, 61–63, 65, 66, 68–70, 72]	“Young women mentioned that they were shy because family planning is considered appropriate only for married people” [58]
					“Some young refugee women noted during interviews that they feel uncomfortable demanding or asking for contraceptives because they are unmarried and many in the camp have conservative social values” [58]
		Communication level	7	[46, 47, 53, 56, 60, 65, 72]	“Heightened incidents of family disputes and breakdowns in family communication and trust that may result in increased gender-based violence” [60]
	Peer pressure		2	[46, 56]	“Some had succumbed to such pressures [from community] even when they understood the risks and the consequences” [56]
	Social exclusion	Stigma and shame	12	[43, 44, 48, 51, 53, 58, 61, 62, 64, 65, 68, 70]	“Girls reported not registering their new-born children for fear of social stigma, and as a consequence forfeiting their entitlement to supplementary food rations provided to mothers and babies. Many young mothers were rumoured to be sharing their food allocations with their babies” [62]
					“I didn’t use it [condom] the last time I had sex because I didn’t want to buy it in the camp. I don’t want anyone to think I’m a prostitute … the last time I went to buy condom and pills in a chemist in the camp; I was embarrassed by some women. They asked me what a young girl like me wanted to do with condom and pills. I was so ashamed that I had to lie that someone sent me” [58]
	Social support	Support and trust	13	[43, 47, 49, 51–55, 57, 58, 67, 70, 71]	“Participants in this study said it was difficult to talk with their parents about sex. They frequently referred to cultural and religious expectations of abstinence from sex prior to marriage, and said that parents are unable to respond to questions and concerns without being judgmental” [53]
					“Lacking a durable network of social relationships led informants to experience a sense of isolation and loss at the time of discovering and resolving their pregnancy” [52]
	Gender equity		1	[59]	“Culture within Kobe camp was described to be highly divided along traditional roles for males and females (…) cultural patterns persist that reinforce inequitable relations between boys and girls in early adolescence” [59]
**Institutional and health system**	Basic principles	Confidentiality and privacy	2	[51, 53]	“While language barriers in health care settings are addressed through interpreter and advocacy services, these services are not readily utilized by young people for sexual health issues due to concerns around confidentiality” [53]
		Cultural competence	5	[53, 61, 62, 68, 70]	“Male teachers are not mostly aware of, or understand the importance of menstrual hygiene management” [70]
	Services	Provision of appropriate services	8	[53, 60, 62, 64, 67–69, 71]	“Adolescent boys with disabilities and caregivers further lamented inequitable services. They said: ‘The staff always serve people they know” [64]
		Resourcing of services	10	[43, 45, 49, 51, 61, 62, 67, 70, 72]	“The condom dispensers in the camps—which allow for confidential use—were reportedly often empty” [45]
	Provider support and tools		6	[52, 53, 64, 66–68]	“A few Somali adolescent girls shared that, ‘They [health providers] will look down upon us because we are disabled,’ or that health providers ‘are harsh’ with them” [64]
	Education		4	[43, 53, 59, 62]	“Very few young people had participated in sexual health education in formal settings, such as schools or education programs in refugee camps. Those who had received some form of education indicated that the focus was on medical aspects of being sexually active, including messages that sex is a cause of disease, and abstinence and condoms are a means of protecting against STIs and unplanned pregnancy” [53]
**Structural**	Infrastructure and security		15	[44, 47, 51–53, 58–60, 62, 64, 69–72]	“Gaining access to different modern contraceptive methods was a challenge for refugees in the camp” [58]
					“The girls indicated they feel safe only inside their homes and generally unsafe outside around their homes, in route to communal locations inside the camp and especially on the way to collect firewood or to find work to supplement household incomes outside the camp” [60]
	Transport		4	[53, 57, 60, 67]	“Young people also identified structural and administrative obstacles such as lack of transport to attend health services, and not knowing how or where to book an appointment” [53]
	Regulations	Public policy and laws	2	[51, 62]	“Girls face several challenges reporting rape and other forms of gender-based violence through formal channels” [51]
		Political context and priorities	4	[53, 56, 62, 68]	“In Cox’s Bazar, facility assessments revealed that unmarried adolescents could not receive contraceptives from camp clinics since commodity distribution is reported to the government” [68]
		Enforcement of laws	2	[62, 71]	“Discussion focused on how best to report rape and other abuse. Neither the police nor church personnel were seen as adequate, as girls were apt to be sexually violated by both” [71]

**Table 3:** Facilitators for SRHR for young refugees.

Category	Sub-category	Code	Number of studies	References	Quotation
**Individual**	Comprehension	Knowledge and information	9	[43, 46, 49, 51, 53, 59, 67, 69, 72]	“Young people said they want to be provided with clear and factual information about STIs, pregnancy and contraception before the onset of sexual relationships” [53]
	Perspectives	Personal beliefs	3	[49, 59, 67]	“Adolescents interviewed, from all three age groups, demonstrated relatively high comfort with body change during puberty” [59]
	Skills and empowerment		3	[43, 64, 71]	“Children felt fairly safe in the camp, and mainly used protection strategies–such as moving about in groups and reporting problems to parents–to address problems encountered outside the camp and in school” [71]
**Social and Community**	Relationship power and expectations		2	[53, 54]	“A committed relationship was described as one where trust was established between partners and there was no ‘playing around” [54, 62]
	Norms	Sociocultural norms	7	[43, 46, 47, 53, 56, 59, 70]	“Many young believe that sexual health promotion and education should be ethno-sensitive rather than ethno-specific” [53]
		Communication level	6	[46, 47, 60, 62, 69, 70]	“All participants agreed that with time and access to information attitudes towards talking about sexual health related matters were slowly changing and there was a growing intergenerational acceptance that families needed to talk and access such information early within their resettlement experience” [46]
	Social support	Social networks	5	[47, 59, 61, 69, 72]	“Mothers are important current sources of information, while adolescents report preferences to learning from… friends and siblings in Ethiopia” [72]
		Support and trust	16	[43, 47, 49, 51–54, 56, 57, 59, 61, 66, 69–72]	“Support from teachers during menstruation by providing a sanitary pad if the girls don’t have it and let the girl go home to get a sanitary pad” [70]
					“Many young people reported that they are likely to talk about sex with friends” [53]
	Gender equity		7	[51–53, 58–61]	“Engage men and boys as agents in reducing violent attitudes and behaviour towards women and girls by focusing on the positive, protective roles men can and do play in the lives of their mothers, wives, sisters and daughters” [53]
	Community organisations		7	[43, 53, 54, 58–61, 59]	“Engage men and women community leaders in discussions on girls’ rights, harmful social norms and harmful traditional practices” [53]
**Institutional and health system**	Basic principles	Confidentiality and privacy	4	[45, 60, 62, 71]	“Increase privacy, confidentiality of counselling and non-judgmental and friendly services, in particular for adolescents and unmarried women” [45]
		Cultural competence	8	[45, 46, 48, 53, 60, 64, 70, 71]	“Health fairs were considered key in enhancing HIV prevention, ‘the sexual health information can be dispersed at the annual health fair” [48]
					“Employing interpreters—sign and other languages—and providing equitable [SRH] services were mentioned as practical ways to improve provider-client interactions and service experiences” [64]
	Services	Provision of appropriate services	11	[45, 47, 60, 61, 63, 64, 66, 67, 70–72]	“Programming that aims to address the SRH needs of very young adolescents should consider the continued education of children and adults that inform and influence their decisions and behaviours. This includes service providers.” [72]
		Monitoring and responsiveness of services	4	[60, 63, 64, 66]	“Prioritize outreach to refugees with disabilities who are isolated in their homes—especially to those with intellectual impairments who can be hidden—to better address their needs and to increase their access to up-to-date and accurate SRH information and services” [64].
		Resourcing of services	2	[61, 72]	“Unmarried adolescents can receive condoms from the camp HIV programs in Cox’s Bazar” [68]
	Provider support and tools		10	[44, 47, 48, 50, 53, 62–65, 69]	“Train young women as peer sexual and reproductive health workers” [62]
					“For adolescents with intellectual disabilities, body mapping activities, pictures and models to convey critical SRH information—especially around acceptable touching—may be helpful aids to use in promoting their protection. As well as information on protective strategies” [63]
	Education	Awareness	4	[43, 47, 53, 61]	Following menstrual hygiene management training: “I know how to use sanitary pads, and I also realize that menstruation is a normal thing” [70]
		Curriculum and information	11	[43, 47, 53, 54, 59, 61, 62, 64, 65, 68, 71]	“After-school sessions during which adolescent girls at the secondary school level discuss issues that directly affect their lives” [61]
		Training	6	[46, 60–62, 68, 70]	“All security enforcement personnel and community leaders who make up traditional justice systems should be trained on prevention of gender-based violence, human rights and child protection, including protecting survivors’ confidentiality” [60]
					“Sensitize male security guards on dealing with child/girl protection issues and the need for confidentiality in reporting incidents” [62]
**Structural**	Infrastructure and security		12	[46, 47, 51, 59–62, 64, 65, 69–71]	“A signing Somali adolescent boy offered more nuanced feedback: ‘It is safe if it has a lock inside. It is unsafe when it doesn’t have a lock, and someone can rape you” [64]
					“Social media and technology …these forms of communication technology were identified as a useful source of sexual health information that negated the embarrassing need to raise the topic with parents or others who might disapprove” [46]
	Regulations	Public policy and laws	3	[46, 51, 71]	“Create an accountability structure in the schools so that teachers, administrators and students all have clear boundaries, and roles and responsibilities are delineated with an appropriate feedback loop in place” [51]
		Enforcement of laws	2	[54, 62]	“Register all children born to refugee adolescent girl mothers and ensure that they receive allocated food and non-food rations for their children” [62]

### Refugee camps or alternatives to camps

#### Individual

Ten publications reported comprehension of SRHR as both a barrier and a facilitator [44, 46, 47, 49, 51, 53, 67, 69, 72]. For example, one publication described linguistic barriers, including a lack of sign interpreters, faced by adolescent males and females in a Kenyan refugee camp [64]. Another study conducted with young females in two Thai refugee camps found that many lacked knowledge about menstruation and the fact that first sex could result in pregnancy [43, 72].

Four publications highlighted the role of skills and empowerment for young people in camps [43, 64, 67, 71]. For example, a qualitative study conducted in a Zimbabwean refugee camp found that young refugees aged 10 to 17 years used protection strategies such as moving in groups [71]. In addition, adolescents with disabilities in a Kenyan refugee camp requested to be taught empowerment-based activities to increase their autonomy and decision-making in relation to SRHR [64].

Six publications reported on risk-taking behaviours in refugee camps [43, 45, 51, 58, 60, 62], such as not using condoms to prevent STIs and unintended pregnancies, due to financial reasons and lack of decision-making power [45, 58]. One publication from Uganda reported on young female refugees engaging in transactional sex to support basic needs, including medical treatment and school fees [51]. Another study conducted in a Malawian refugee camp found that parents encouraged their children to practice transactional sex with older men in exchange for goods or money [71].

#### Social and community

Fourteen publications reported that GBV, including sexual harassment, sexual violence, forced sex, home invasion and rape, exploitation and discrimination occurred frequently in camps and alternatives to camps [43, 44, 47, 51, 59–64, 66, 69, 71, 72]. For example, a mixed methods study conducted in a Ugandan refugee camp described how girls faced high risk of sexual violence while travelling to and from school and the market [51]. Interviews with men and boys from the same camp revealed that sexual harassment often occurred due to male “idleness and boredom”. The same Ugandan study also highlighted the role of relationship power and expectations, where teachers in a refugee camp abused their authoritative role by requesting sexual favours of girls and using corporal punishment if they refused [51]. Thirteen studies identified lack of social support and trust as a barrier to SRHR [43, 47, 49, 51–55, 57, 58, 67, 70, 71], for example, a grey report from a Tanzanian refugee camp that described male teachers accusing girls of lying about menstrual cramps [70].

In terms of facilitators, three studies conducted in Tanzanian and Ethiopian refugee camps identified the value of supportive community organisations for SRHR. In particular, training of community leaders on child protection and confidentiality for survivors of GBV was considered a facilitator [59, 60, 62]. Three studies conducted in Ethiopia, Lebanon, Uganda and Tanzania also recommended working with parents and teachers to discuss issues such as menstrual hygiene management and the prevention of child marriage [61, 69, 72].

#### Institutional and health system

Two studies reported lack of confidentiality and adolescent-friendly health services as barriers to SRHR [51, 53]. For example, a study in a Ugandan refugee camp noted that while antenatal, mental health and HIV-related services were available, these services were not designed for young people, which discouraged them from utilising services and confiding in providers [45]. Publications also recommended increased privacy and confidentiality [45, 60, 62, 71], employing interpreters [71], and ensuring age-appropriate services (e.g. access to contraception) [60] in a safe space [62] as potential facilitators. Ten studies conducted in countries such as Kenya, Ethiopia and Nepal further identified provider support and tools as facilitators to SRHR [44, 47, 48, 50, 53, 62–65, 69], including the training of health service providers to respond to the specific SRHR needs of young refugees with disabilities [64].

#### Structural

Twelve publications reported that inadequate infrastructure and security were barriers to young refugees’ SRHR when residing in refugee camps or alternatives to camps [44, 47, 51, 58–60, 62, 64, 69–72]. Examples included insecure shelters, lack of lighting [62], unsafe latrines [64], lack of water, sanitation and hygiene, and lack of access to menstrual hygiene management facilities in camps and schools [70]. In addition, five publications that examined refugee camps in sub-Saharan Africa reported that young refugees, particularly girls, felt unsafe outdoors when collecting firewood and water, noting lack of adequate policing [51, 60, 62, 69, 71].

Correspondingly, 10 publications discussed effective camp management as a key facilitator for SRHR [46, 51, 60–62, 64, 65, 69–71]. For example, the provision of adequate lighting [51, 62], increased security [51, 60, 62, 71], prevention of alcohol and marijuana use [71], ensuring latrines and houses have sturdy doors and secure locks [62, 64], and building of female-friendly latrines (i.e. with buckets for menstrual hygiene management) in schools, around the camp and in the dwellings of single girl-headed households [62, 70]. It was also suggested that charcoal be distributed throughout the camp to ensure children would not have to collect firewood alone and risk exposure to violence and abuse [71].

Finally, six studies in different refugee camps reported structural regulations such as public policy and laws [51, 62], political context and priorities [53, 56, 62, 68] and lack of law enforcement [62, 71] as barriers to SRHR. For example, a study conducted in a Zambian refugee settlement found that children did not feel comfortable reporting GBV to the police or church due to fear of being sexually assaulted by both [71]. Other barriers included restricted access to contraception for unmarried adolescent refugees [68] and the illegal status of abortion services in Uganda [51].

### Resettled to a third country

#### Individual

Similar to what was found in refugee camps, nine publications reported low awareness of SRHR among refugees who had been resettled to a third country such as Australia or the United Kingdom [46, 52–57]. Three publications reported that young refugees only received SRHR information for the first time after having contracted an STI or becoming pregnant after resettling in Australia [46, 55, 56]. One publication reported on language barriers in the new country as a barrier to SRHR [53].

Three publications identified poor risk perceptions and misunderstandings as key barriers to SRHR for refugees who had been resettled [46, 54, 56]. For instance, in Australia, many refugees did not see themselves at risk of HIV, as they thought the health screening process prior to entry to the country prohibited the admission of any people with HIV. Furthermore, while HIV was regarded as terminal, many young refugees did not consider it a health concern in Australia [56]. Similarly, another Australian study conducted with resettled young refugees found that many young women expressed fear over abortions due to knowledge about unsafe procedures that had occurred in refugee camps while they were in transit [57].

#### Social and community

Two publications that studied resettled young refugees identified a disparity between the norms of a refugee’s country of origin and the norms of their host country [46, 56], for example, parents reported being worried about the liberal norms and values in Australia and associated SRHR knowledge with promiscuity [46, 56]. Parents also expressed concerns about the content included in sexuality education curricula [50]. These differing sociocultural beliefs around SRHR resulted in intergenerational conflict within families [48].

In line with this, twelve publications described how addressing sociocultural norms [43, 46, 47, 53, 56, 59, 70] and improving communication between young people and parents [46, 47, 60, 62, 69, 70] can facilitate the SRHR of young refugees. For instance, an open attitude towards talking about sex assisted young people and their families to navigate the differing sociocultural attitudes they faced after resettling in Australia, thereby reducing some of the intergenerational confusion and conflict [46, 53]. Similarly, one publication that studied African-born refugees in the United States recommended the engagement of parents, community and religious leaders to assist with HIV-prevention efforts [48].

#### Institutional and health system

A lack of provider support was identified as a barrier by young refugees who had been resettled to Australia and the United Kingdom [52, 53]. For example, young pregnant women reported that they encountered stereotyped judgements from health professionals in the United Kingdom, which made them feel like they were not in control of their childbirth [52]. Provider support and tools were identified as facilitators by young refugees who had been resettled to Australia and the United States [48, 53], including the support of doctors and other health providers in providing information on SRH [53]. In addition, one publication from Australia emphasised that sexual health education for refugees needs to be interactive, using activity-based learning [53].

#### Structural

Three publications reported that refugees felt constrained by resettlement needs such as finding housing, a job and navigating their new society, and therefore could not prioritise their health, including SRH [51, 53, 56]. However, social media and technology were reported as facilitators in one publication on Sudanese refugees who had been resettled to Australia as they found these communication channels to be safe, confidential sources of sexual health information [46].

### Interventions for the SRHR of young people in refugee contexts

In addition to describing facilitators and barriers, nine publications also reported on specific interventions to improve SRHR among young refugees [50, 51, 61, 63, 65, 68–71]. Table 4 presents a detailed overview of these interventions according to the type of intervention, setting, population, outcome and methods. Overall, interventions tended to be educational projects within refugee camps aimed to teach young people about SRHR, in addition to projects that involved the sensitisation or training of peer educators, religious leaders, parents and service providers [50, 65, 69, 71]. For example, a school-based intervention in a Rwandan refugee camp used weekly educational sessions facilitated by community health educators on reproductive health at primary and secondary schools. By expanding the community health educators’ role to counsel adolescents on family planning, the communities’ comfort and acceptance of the programme was improved [65]. Another intervention trained 60 young men to identify, address and prevent GBV because they were considered to be more receptive than older men to learn about GBV and address gender norms [50].

**Table 4:** Summary of the identified SRHR interventions.

	Title	Type of intervention	Intervention setting	Population and age	Outcomes	Supplementary information
1	“It doesn’t matter if you are disabled. You are talented”—The intersection of sexual and reproductive health and disability for Bhutanese refugees in Damak, Nepal [63]	Community-based awareness-raising project	Refugee camp	Males and females (15–19 years)	Implemented monthly awareness-raising activities	Included different stakeholders in the process (including disability centres)
					Equipped youth friendly centre libraries with SRH related book	
					Provided consultation hours on SRH	
2	Adolescent Sexual and Reproductive Health Programs in Humanitarian Settings: An In-depth Look at Family Planning Services [65]	School-based educational project	Refugee camp	Males and females (10–19 years)	Activities implemented once a week on reproductive health at primary and secondary schools	Staff moved systematically through the school classrooms, shifting the focus each week to ensure the entire school was covered by the end of the year
					Health educators led adolescent sexual and reproductive health sensitisation activities in each class	Incorporated nurses from the health facility as a way to engage in-school adolescents in the health care system
					Educators and providers were selected from the community in a manner that built support for the program	Before the program, community members, religious leaders and groups of women and youths were convened for education activities related to the planned program
					Many family planning providers were identified for the program, since they had previously working as counsellors	Expanding their role to counsel adolescents on family planning improved the communities’ comfort and acceptance of the program
					Peer educators were selected from different beneficiary populations, ensuring representation from both genders and from refugee populations targeted by this intervention	Working from what the community was ready to accept was one of the key points of learning from this program
3	Adolescent Refugees and Migrants: A Reproductive Health Emergency [50]	Youth training, community sensitisation activities	Refugee camp	Males and females (10–19 years)	Frequent group education activities were held for youth leaders, peer educators, teachers, religious leaders, parents and service providers to sensitise them and reduce community opposition to providing reproductive health services to youth	Peer educators provided referrals for health services and distributed condoms and informational materials to other youth
					Trained young male camp residents to identify, address, and prevent gender-based violence and improve the reproductive health of young men and women	
4	Family planning in refugee settings: findings and actions from a multi-country study [68]	Youth training	Refugee camp	Males and females (15–59 years)	Refugee youth trained in family planning–including for emergency contraceptives	Parental attitudes towards contraception use are not fixed—an important consideration for policy development in this area
					Disseminated family planning information through “adolescent corners” in the camps	
5	Menstrual Hygiene Management for Education in Emergencies: A Study for Plan International Tanzania [70]	Menstrual hygiene management training	Refugee camp	Females (10–18 years)	Distributed ‘dignity kits’ to adolescent refugee girls; these kits included an “AfriPad”, a sanitary pad that can be washed, dried and re-used for 12 or more months	Suggested the provision of MHM trainings, gender-friendly latrines, and sanitary pads would establish a relatively more gender-friendly environment
					Menstrual hygiene management training was provided to girls attending the school	
6	Tanzania: Girls Clubs Essential to Equity in Education [61]	Menstrual hygiene management training	Refugee camp	Females (10–19 years)	Established Girls Clubs to respond to basic needs, including providing menstrual hygiene items, regular hygiene items	Suggested expanding Girls Clubs to reach more girls, including those in upper primary classes
7	A Safe Place to Shine: Creating Opportunities and Raising Voices of Adolescent Girls in Humanitarian Settings [69]	Provider training	Refugee camp	Females (13–19 years)	Trained health and gender-based violence case management service providers to improve their attitudes towards adolescent girls and the quality of support they provide to girls	Programme provided gender-based violence case management services, which included referring adolescent girls who experienced violence to appropriate services
					Staff used standardised materials to train service providers, making modifications to ensure they were relevant to adolescent girls	
8	Through the eyes of a child: refugee children speak about violence [71]	Youth training, community workshops	Refugee camp	Males and females (15–19 years)	Peer education on life-skills, sports and cultural activities and sessions on gender-based violence and conflict resolution were held at the youth centre for both refugee and local children	Child-friendly spaces
					Workshop held with community development and health workers, teachers, police and others to encourage discussion on how to address gender-based violence in the camp	Gender-based violence was addressed in schools, during HIV education, drama and poetry groups; workshop was held on case referral
					Workshop held on the topic of referrals in gender-based violence cases	Limited funding prevented initiation of income-generating activities for girls to prevent transactional sex
9	Scattered Dreams, Broken Promises: An Assessment of the Links between Girls’ Empowerment and Gender-based Violence in the Kyaka II Refugee Settlement, Uganda [51]	Confidential reporting channels, staff training	Refugee settlement	Males and females (10–16 years)	Created an accountability structure in the schools so that teachers, administrators and students all have clear boundaries, and roles and responsibilities are delineated with an appropriate feedback loop in place	Suggested the increase of protection in schools, the sensitisation of teachers to stop unfair punishment of children and the fostering of a supportive environment for girls to report gender-based violence

## Discussion

To the best of our knowledge, this scoping review is the first to map what has been reported on perceived barriers and facilitators, as well as interventions, related to SRHR for young refugees at a global level. We found that most publications reported on barriers, rather than facilitators, including lack of knowledge and information, GBV, sociocultural norms, stigma and shame, lack of social support, and lack of infrastructure and security.

At first glance, many of these barriers to SRHR are similar to those faced by other young people, including stigma and shame surrounding young people’s sexual activity [43, 44, 48, 51, 53, 58, 61, 62, 64, 65, 68, 70], lack of adolescent-friendly services [4] and the importance of confidentiality [24, 73–75]. However, our review indicates that many barriers are exacerbated by the refugee context. Deficiencies in infrastructure and security (e.g. insecure shelters, lack of lighting, unsafe latrines), financial dependency, poor awareness of SRHR and lack of protection for unaccompanied minors, likely increase the risk of sexual abuse, exploitation and other types of GBV. Poor infrastructure for water, sanitation and hygiene, and lack of confidential, respectful and adolescent-friendly SRH services in many refugee camps and alternatives to camps also limit young people’s access to menstrual hygiene, contraception, STI prevention, SRH care and support.

However, not all publications evaluated or identified facilitators or interventions in relation to the reported barriers. While the most commonly reported facilitators (e.g. social support and trust, provision of appropriate services, provider support and tools, improved safety) directly tackle some challenges, we did not identify any facilitators to address peer pressure, social exclusion or stigma for young refugees. In particular, very few studies addressed conflicting sociocultural beliefs related to individual versus family norms which can play a central role when young people transition through puberty and adolescence [7, 76], leaving a gap in the literature.

Only nine publications described SRHR interventions for young refugees [50, 51, 61, 63, 65, 68–71], with most involving service provision and/or training of youth, peer educators, parents, community members, religious leaders and providers (e.g. GBV prevention and response). While an assessment of the evidence and effectiveness of these interventions is beyond the current scoping review, we found that all interventions identified were only conducted in refugee camps or settlements in countries such as Nepal, Tanzania and Uganda. While the under-reporting of interventions is possible, this still indicates that more research is needed on what types of interventions are most beneficial for different groups of young refugees across different contexts.

Young refugees are a highly heterogeneous population–yet, most publications did not present disaggregated data by characteristics such as age, sex, sexual orientation, race/ethnicity, socioeconomic background or physical and psychological impairments. Four publications specifically referred to young refugees with disabilities [63, 64, 66, 67], noting the importance of training service providers on how to respectfully communicate with these refugees and understand and meet their SRHR needs. While a lack of disaggregated data is not unique to refugee populations, such data is critical to understand and tailor programs to meet the needs of underrepresented sub-groups. In particular, we found no publications on young lesbian, gay, bisexual, transgender, queer or intersex refugees, despite the fact that this population faces a disproportionately high risk of HIV and STIs due to stigma, legal context, financial vulnerability, poor mental health and lack of services [77].

Furthermore, we found that little attention is given to the sexual and reproductive rights of young refugees in the literature. While several publications focus on GBV, few discuss sexual rights including an individual’s right to choose if, when and with whom to be in a relationship and have sex, as well as reproductive rights related to abortion. This conclusion is supported by Hartmann et al. who found that while many SRH interventions address gender inequalities, very few involve rights [78]. As suggested by Orza et al., the lack of a rights-based approach in interventions may be because many of these rights, sexual rights in particular, have not been recognised in international human rights conventions and in the Sustainable Development Goals, which could reinforce underlying inequalities [79]. From a life course perspective, it is critical to prioritise young refugees’ sexual and reproductive rights through adolescence and into adulthood as denial of these rights will put them at high risk of GBV, unintended pregnancies, unsafe abortions, and preventable maternal deaths. Interventions should focus on building capacity among local actors and authorities, including camp health service providers, community leaders and young people themselves so they are able to support and protect the lives, health and dignity of girls, boys and non-binary individuals in refugee contexts.

### Strengths and limitations

This scoping review highlights SRHR barriers and facilitators for young refugees globally and identifies major research gaps. Key strengths include: the scoping approach, which allowed the mapping of literature across different countries and refugee contexts while drawing on diverse methodologies; the fact that most data came from young refugees themselves; the use of the socio-ecological framework to synthesise the data; the critical appraisal of all primary studies; and the presentation of findings by type of refugee context (i.e. camps or alternatives to camps versus resettled to a third country). Our aim was to establish a knowledge base and identify knowledge gaps, rather than merely comparing and contrasting findings across different settings. Even though we strived for a comprehensive search, it is possible that we missed relevant studies as we restricted the review to publications written in English within the last 10 years. During initial revisions of the manuscript, we noted that the inclusion of certain filters (e.g. Humans) in the original PubMed search resulted in the omission of publications that had not yet been indexed. We therefore removed the filters and re-conducted the original PubMed search with the same date restrictions, resulting in the inclusion of five additional publications. Furthermore, few publications reported results disaggregated by age and sex, undermining efforts to conduct stratified analyses to better understand the needs of specific sub-groups. Finally, due to the cross-sectional design of many studies, it is possible that some of the reported barriers and facilitators to SRHR were experienced before the study participants became refugees.

Our review identified several avenues for future research, such as institutional and structural facilitators (e.g. health services, the role of laws and policies) for resettled young refugees where literature is currently missing. In addition, further research could differentiate between experiences faced by younger versus older adolescents, and males versus females to support age and gender perspectives in analyses. More evidence is needed on facilitators and effective interventions, particularly for young refugees who have been resettled. Future research could also investigate whether any of the identified barriers, facilitators or interventions are applicable in refugee settings other than those included in the current review. In addition, as no publications reported on SRHR barriers and facilitators for young lesbian, gay, bisexual, transgender, queer or intersex refugees, this points to a large gap in the literature to be addressed in future studies and interventions.

## Conclusions

In the foreseeable future, war and civil unrest, poverty and climate change will continue to forcibly displace people from their countries of origin. Our results highlight that young refugees are among the world’s most vulnerable populations and have specific SRHR needs. While they experience similar barriers to SRHR as other young people, many of these are exacerbated by the refugee context, such as the high risk of sexual abuse and exploitation as well as other forms of GBV. It is crucial that effective interventions are implemented to improve SRHR outcomes for all refugees, with a particular focus on the safety and security of young people. Addressing these needs will help young men, women and non-binary individuals in refugee contexts to live healthy and productive lives now and in the future. Despite international commitments to ensure young refugees’ SRHR, such as the Global Strategy for Women’s, Children’s and Adolescents’ Health, few interventions actually address their specific needs–calling for further investment and commitment from the global health community to strengthen and protect the SRHR of young people in refugee contexts.

## Supporting information

S1 Appendix Review protocol. (PDF)

S2 Appendix PRISMA extension for scoping reviews checklist. (PDF)

S3 Appendix Inclusion criteria. (PDF)

S4 Appendix Keywords used for searching. (PDF)

S5 Appendix Full search strategy used for the ‘Global Health’ electronic database. (PDF)

S6 Appendix 7-item form for the screening process. (PDF)

S7 Appendix 27-item template for data extraction. (PDF)

S8 Appendix Examples of the coding process. (PDF)

S9 Appendix Critical appraisal of included publications. (PDF)
