# Supplementary material for: Barriers and facilitators for the sexual and reproductive health and rights of young people in refugee contexts globally: A scoping review
Source: PLoS One. 2020 Jul 20;15(7):e0236316. doi: 10.1371/journal.pone.0236316 (PMC7371179; doi:10.1371/journal.pone.0236316)
Supplement: S1 Appendix — (PDF) [file pone.0236316.s001.pdf]

S1 Appendix. Review protocol.

Title

Barriers and facilitators to sexual and reproductive health and rights for young people in refugee contexts globally: A systematic scoping review

Background

In order to improve sexual and reproductive health and rights (SRHR) in the context of conflict and displacement, there is an urgent need to gather information to ensure that the needs of young refugees are being met effectively and appropriately. The available literature tends to report on the SRHR needs of young people or on the SRHR needs of refugees in general, but there is limited information available on the SRHR of young refugees. To our knowledge, no study has compiled the literature on barriers and facilitators to SRHR for 10- to 24-year-old refugees, and the SRHR interventions that target this population. Therefore, this review could help identify gaps and opportunities for tailoring SRHR programmes to meet the specific needs of young refugees in different settings (e.g. refugee camp vs. residing in a host country).

Aim

To identify and synthesise the current literature on perceived barriers and facilitators to SRHR among young refugees globally, and the interventions created to address their needs.

Research questions

1. What has been reported on the perceived barriers and facilitators to sexual and reproductive health and rights for young refugees worldwide?
2. What has been reported on current sexual and reproductive health and rights interventions that target young refugees?

Methodology

Design

As this is a relatively new area of research, a scoping review study design has been chosen to establish and map what literature is emerging. This scoping review will examine evidence using a method based on a number of scoping review guidelines. These include the procedure devised by Arksey and O'Malley, and the enhancements to this procedure suggested by Levac,

Colquhoun and O'Brien. The method will also draw upon Joanna Briggs Institute's approach to conducting scoping reviews.

Study setting

This scoping review will consider all applicable publications, regardless of their country of origin. As this is a new area of research, there are a limited number of resources available. Therefore, the search will not be restricted further by filtering by geographical region. However, as this review will focus on people who have fled their country of origin and are unable or unwilling to return because of well-established fear of persecution, studies will not be applicable if they address migrant workers or if they address internal migrants who have moved from one part of a country to another part of the same country.

Sampling strategy

For the selection of articles, this project will follow the PRISMA flow diagram for scoping reviews. This includes four stages:

1. Identification – records will be identified through database searching and manual searching, and duplicates will be removed;
2. Screening – titles and abstracts will be screened, and records will be included or excluded according to the inclusion criteria;
3. Eligibility – full-text articles will be assessed for eligibility, and those with reason to be excluded will be excluded;
4. Inclusion – the remaining studies will be included.

Inclusion criteria

An article or publication will be included for full-text screening if it meets the following criteria:

1. The article or publication reports on:
    a. barriers and/or facilitators to sexual and reproductive health and/or rights; or
    b. a sexual and reproductive health and/or rights intervention;
2. The study population includes:
    c. young people (aged 10–24); and
    d. asylum seekers, refugees and/or migrants who have moved from one country to another;

3. The article or publication is written in English; and
4. The article or publication was published in the last 10 years (2008 onwards).

The United Nations Children's Fund, the United Nations Population Fund and the World Health Organization recognise 10-24-year-olds as ‘young people’. This age frame has been chosen because it is all-encompassing and allows for the inclusion of studies that look at ‘adolescents’ (10-19-year-olds), ‘youth’ (15-24-year-olds), or people across these age frames.

In order to ensure relevance, only studies from 2008 onwards will be included (search to be completed on 15 February 2018). This will make the study period range over 10 years.

Data collection/search strategy for identification of studies

Electronic databases searching:

The following electronic databases will be searched for peer-reviewed articles: PubMed and Global Health.

The following electronic databases will be searched for grey literature: POPLINE.

Manual searching:

Reference lists from identified studies will be searched manually.

Literature from the following international organisations will be hand searched for relevant information: UNFPA, WHO, UNICEF, Guttmacher Institute, Women’s Refugee Commission and PLAN International. These six international organisations were selected for hand searching as they are known to the authors as having engaged in research and advocacy for SRHR and/or in humanitarian settings. These particular organisations have been selected to augment the collection of papers identified by the electronic searches.

Quality assessment

The quality of the included publications will be assessed using the Cochrane Collaboration Qualitative Methods Group’s criteria for the critical appraisal of qualitative research and the Effective Public Health Practice Project’s Quality Assessment Tool for the quantitative sections of the mixed methods publications.

Planned analyses

The included publications will be analysed using qualitative content analysis. This method allows for the systematic analysis of the messages found in any type of communication, and this will be appropriate considering the different types of publications analysed in scoping reviews. The analysis will focus on manifest content. This will involve describing the obvious content, instead of focusing on latent content and interpreting the underlying meaning of the text. We will conduct a charting process to sort and organise the included publications and elicit information and insights relevant to our review, focusing on the study region, setting, sample size and participants. Findings will be synthesised using a thematic, narrative approach.

Ethical considerations

A scoping review does not require ethical approval as it examines data from the existing literature that is available. However, ethical considerations will be made as this area of research requires ethical sensitivity. During the full-text screening, all potentially applicable publications will be examined to ensure they received ethical approval. Publications that involved participants will also be checked to ensure they received informed consent from all participants.

Dissemination

We will aim to publish a paper in an appropriate journal.
