# Supplementary material for: Barriers and facilitators for the sexual and reproductive health and rights of young people in refugee contexts globally: A scoping review
Source: PLoS One. 2020 Jul 20;15(7):e0236316. doi: 10.1371/journal.pone.0236316 (PMC7371179; doi:10.1371/journal.pone.0236316)
Supplement: S3 Appendix — (PDF) [file pone.0236316.s003.pdf]

S3 Appendix. Inclusion criteria.

1. The article or publication reports on:
    a. barriers and/or facilitators to sexual and reproductive health and/or rights; or
    b. a sexual and reproductive health and/or rights intervention;
2. The study population includes:
    c. young people (aged 10–24); and
    d. refugees, asylum seekers and/or persons forced to move from one country to another;
3. The article or publication is written in English; and
4. The article or publication was published in the last 10 years (2008 onwards).
