# Supplementary material for: Barriers and facilitators for the sexual and reproductive health and rights of young people in refugee contexts globally: A scoping review
Source: PLoS One. 2020 Jul 20;15(7):e0236316. doi: 10.1371/journal.pone.0236316 (PMC7371179; doi:10.1371/journal.pone.0236316)
Supplement: S4 Appendix — (PDF) [file pone.0236316.s004.pdf]

S4 Appendix. Keywords used for searching.

1. "sexual health" OR "sexual rights" OR "reproductive health" OR "reproductive rights" OR SRHR OR SRH OR contracepti* OR pregnan* OR "maternal health" OR maternity OR antenatal OR postnatal OR obstetric OR delivery OR aborti* OR post-aborti* OR "family planning" OR "sexually transmitted" OR STI OR STD OR HIV OR "gender-based violence" OR "intimate partner violence" OR "sexual abuse" OR "female genital" OR FGM OR "female circumcision"
2. AND (barrier* OR facilitator* OR intervention* OR service* OR program* OR information OR education*)
3. AND (adolescen* OR young OR youth)
4. AND (migrant OR migrants OR migration OR refugee* OR "asylum seekers")
