# Supplementary material for: Barriers and facilitators for the sexual and reproductive health and rights of young people in refugee contexts globally: A scoping review
Source: PLoS One. 2020 Jul 20;15(7):e0236316. doi: 10.1371/journal.pone.0236316 (PMC7371179; doi:10.1371/journal.pone.0236316)
Supplement: S5 Appendix — (PDF) [file pone.0236316.s005.pdf]

S5 Appendix. Full search strategy used for the 'Global Health' electronic database.

**1) Sexual and Reproductive Health and Rights**	
Keywords:	"sexual health" OR "sexual rights" OR "reproductive health" OR "reproductive rights" OR SRHR OR SRH OR contracepti* OR pregnan* OR "maternal health" OR maternity OR antenatal OR postnatal OR obstetric OR delivery OR aborti* OR post-aborti* OR "family planning" OR "sexually transmitted" OR STI OR STD OR HIV OR "gender-based violence" OR "intimate partner violence" OR "sexual abuse" OR "female genital" OR FGM OR "female circumcision"
Controlled vocabulary:	sexual health/ OR reproductive health/ OR contraceptives/ OR contraception/ OR pregnancy/ OR pregnant adolescents/ OR maternity/ OR prenatal care/ obstetrics/ OR abortion/ OR induced abortion/ OR family planning/ OR sexually transmitted diseases/ OR human immunodeficiency viruses/ OR HIV infections/ OR domestic violence/ OR spouse abuse/ OR sexual abuse/
**AND**	
**2) Barriers/Facilitators/Interventions**	
Keywords:	barrier* OR facilitator* OR intervention* OR service* OR program* OR information OR education*
Controlled vocabulary:	social barriers/ OR intervention/ OR health services/ OR maternity services/ OR medical services/ OR public health services/ OR services/ OR social services/ OR programs/ OR program evaluation/ OR information/ OR education/ OR education programmes/ OR health education/ OR medical education/ OR sex education/ OR prenatal education/
**AND**	
**3) Population: Young People (10-24 years)**	
Keywords:	adolescen* OR young OR youth
Controlled vocabulary:	adolescents/ OR youth/
**AND**	
**4) Population: Refugees, Asylum Seekers**	
Keywords:	migrant OR migrants OR migration OR refugee* OR "asylum seekers"
Controlled vocabulary:	migrants/ OR migration/ OR refugees/

Filters: English Language, Publication Year (2008-current)
