# Supplementary material for: Barriers and facilitators for the sexual and reproductive health and rights of young people in refugee contexts globally: A scoping review
Source: PLoS One. 2020 Jul 20;15(7):e0236316. doi: 10.1371/journal.pone.0236316 (PMC7371179; doi:10.1371/journal.pone.0236316)
Supplement: S6 Appendix — (PDF) [file pone.0236316.s006.pdf]

S6 Appendix. 7-item form for the screening process.

Background information:<br>Paper identification #<br>Name of screener<br>Date of screening<br>Does the title fit?
Screening questions (1 indicates 'yes' and 0 indicates 'no'):<br>Is the study published in a peer-reviewed journal?<br>If "No" to previous, is the study published in the grey literature?<br>Is the study about perceived barriers/facilitators to SRHR?<br>Is the study about a SRHR programme/intervention?<br>Are the participants/people in focus aged between 10-24?<br>Are the participants/people in focus refugees or people who have had to move from one country to another?<br>Has the study been published in the last 10 years (2008-onwards)?
Assessment:<br>Included<br>Excluded following initial screening (Title/Abstract)<br>Excluded following full text screening<br>If excluded, please indicate the reason why:
