# Supplementary material for: Barriers and facilitators for the sexual and reproductive health and rights of young people in refugee contexts globally: A scoping review
Source: PLoS One. 2020 Jul 20;15(7):e0236316. doi: 10.1371/journal.pone.0236316 (PMC7371179; doi:10.1371/journal.pone.0236316)
Supplement: S7 Appendix — (PDF) [file pone.0236316.s007.pdf]

S7 Appendix. 27-item template for data extraction.

Background information:<br>Paper identification #<br>Name of data extractor<br>Date of extraction (dd/mm/yy)<br>Publication title<br>Type of publication (grey literature or published literature)<br>Year of publication<br>Authors names<br>Journal name<br>Institution (for grey literature)<br>Country<br>Language<br>Population<br>Age range of participants<br>Gender (M/F/mixed)<br>Ethical considerations made/ethical approval and consent received<br>Other important participant characteristics
Study design and analysis:<br>Study design<br>Purpose of data collection<br>Sample size<br>Data collection methods (e.g. focus group discussions)<br>Data analysis techniques<br>Key findings
Barriers:<br>Barriers
Facilitators:<br>Facilitators<br>Other important details about barriers and/or facilitators
Interventions:<br>Intervention name<br>Intervention size (# of participants)<br>Main intervention setting (e.g. school)<br>Activities<br>Other important details
