# Supplementary material for: Barriers and facilitators for the sexual and reproductive health and rights of young people in refugee contexts globally: A scoping review
Source: PLoS One. 2020 Jul 20;15(7):e0236316. doi: 10.1371/journal.pone.0236316 (PMC7371179; doi:10.1371/journal.pone.0236316)
Supplement: S8 Appendix — (PDF) [file pone.0236316.s008.pdf]

S6 Appendix. Examples of the coding process.

Meaning Unit	Condensed Meaning Unit	Code	Sub-category	Category
'Girls face several challenges reporting rape and other forms of GBV through formal channels' [43]	Challenges reporting rape and GBV	Public policy and laws	Regulations	Structural
'The condom dispensers in the camps—which allow for confidential use—were reportedly often empty' [39]	Condom dispensers in camps often empty	Resourcing of services	Services	Institutional/ health system
