# Supplementary material for: Barriers and facilitators for the sexual and reproductive health and rights of young people in refugee contexts globally: A scoping review
Source: PLoS One. 2020 Jul 20;15(7):e0236316. doi: 10.1371/journal.pone.0236316 (PMC7371179; doi:10.1371/journal.pone.0236316)
Supplement: S9 Appendix — (PDF) [file pone.0236316.s009.pdf]

S9 Appendix. Critical appraisal of included publications.

Paper Identification #	Qualitative Criteria					Quantitative Criteria						
	Credibility	Transferability	Dependability	Confirmability	**Quality Rating**	Selection Bias	Study Design	Confounders	Blinding	Data Collection Methods	Withdrawals and Drop-outs	**Global Rating**
4820954	B	B	A	B	HIGH	N/A	N/A	N/A	N/A	N/A	N/A	N/A
7297284	B	B	B	C	MODERATE	N/A	N/A	N/A	N/A	N/A	N/A	N/A
7297285	B	B	B	C	MODERATE	N/A	N/A	N/A	N/A	N/A	N/A	N/A
7297286	**B**	B	B	C	MODERATE	N/A	N/A	N/A	N/A	N/A	N/A	N/A
14380710	A	A	A	B	HIGH	N/A	N/A	N/A	N/A	N/A	N/A	N/A
14380774	B	A	C	C	MODERATE	N/A	N/A	N/A	N/A	N/A	N/A	N/A
14380876	B	B	C	C	MODERATE	N/A	N/A	N/A	N/A	N/A	N/A	N/A
14380885	B	A	C	C	MODERATE	N/A	N/A	N/A	N/A	N/A	N/A	N/A
14380999	B	A	B	C	MODERATE	2	3	3	3	3	N/A	WEAK
14381070	B	B	B	B	HIGH	N/A	N/A	N/A	N/A	N/A	N/A	N/A
14382433	B	B	B	C	MODERATE	2	3	3	3	2	N/A	WEAK
14382444	B	A	B	B	HIGH	N/A	N/A	N/A	N/A	N/A	N/A	N/A
14382480	C	B	C	C	LOW	2	3	3	3	3	N/A	WEAK
14382490	C	B	C	B	MODERATE	N/A	N/A	N/A	N/A	N/A	N/A	N/A
14382491	B	C	C	C	LOW	2	3	3	3	3	N/A	WEAK
14382492	B	B	B	A	HIGH	3	N/A	N/A	N/A	N/A	N/A	N/A
14382493	B	A	C	C	MODERATE	N/A	N/A	N/A	N/A	N/A	N/A	N/A
14382497	B	A	B	A	HIGH	3	3	3	3	2	N/A	WEAK
14382504	C	B	C	C	LOW	3	3	3	3	2	N/A	WEAK
14382522	B	B	C	C	MODERATE	N/A	N/A	N/A	N/A	N/A	N/A	N/A
14382523	C	B	C	C	LOW	N/A	N/A	N/A	N/A	N/A	N/A	N/A
15375508	B	B	B	C	MODERATE	N/A	N/A	N/A	N/A	N/A	N/A	N/A
10000001	B	A	B	C	MODERATE	2	3	3	3	3	N/A	WEAK
10000002	C	B	C	C	LOW	N/A	N/A	N/A	N/A	N/A	N/A	N/A
10000005	A	A	A	B	HIGH	2	1	1	2	2	3	MODERATE
10009	N/A	N/A	N/A	N/A	N/A	1	3	3	1	3	N/A	WEAK
10010	A	B	A	C	MODERATE	N/A	N/A	N/A	N/A	N/A	N/A	N/A
10012	A	A	A	C	HIGH	N/A	N/A	N/A	N/A	N/A	N/A	N/A
10078	B	A	A	A	HIGH	N/A	N/A	N/A	N/A	N/A	N/A	N/A
10118	B	A	B	C	MODERATE	1	3	1	1	2	N/A	MODERATE
